# Value of a repeat ^18^F-flotufolastat PET scan following a negative scan in patients with suspected biochemical recurrence of prostate cancer after radical prostatectomy

**DOI:** 10.1007/s00259-026-07786-6

**Published:** 2026-02-21

**Authors:** Sonja Kirchhoff, Andreas Lederer, Theo Lorenzini, Michael Gammel, Daniel Sasse, Matthias Heck, Bernhard Haller, Wolfgang A. Weber, Matthias Eiber, Isabel Rauscher

**Affiliations:** 1 Department of Nuclear Medicine, School of Medicine, Technical University of Munich, Ismaninger Straße 22, 81675 Munich, Germany; 2 Clinic and Policlinic for Radiology, Ludwig-Maximilians-University Munich, Munich, Germany; 3 Department of Urology, School of Medicine, Technical University of Munich, Munich, Germany; 4 Department of Urology, Faculty of Medicine, University of Augsburg, Augsburg, Germany; 5 School of Medicine and Health, Institute of AI and Informatics in Medicine, TUM University Hospital, Technical University of Munich, Munich, Germany; 6 Bavarian Cancer Research Center, Erlangen, Germany

**Keywords:** Biochemical recurrence, Hybrid imaging, Positron emission tomography, Prostate cancer, Prostate‐specific membrane antigen

## Abstract

**Purpose:**

To investigate the potential of a second ^18^F-flotufolastat positron emission tomography (PET) scan in patients with suspected biochemical recurrence (BCR) of prostate cancer (PCa) who had a previous negative ^18^F-flotufolastat PET, and to identify prostate-specific antigen (PSA) parameters influencing detection efficacy.

**Methods:**

Our retrospective analysis included data from patients who underwent second ^18^F-flotufolastat PET between 2019 and 2024, following negative ^18^F-flotufolastat PET for suspected BCR of PCa (PSA ≥ 0.2 ng/mL) after radical prostatectomy. Two board-certified experts classified ^18^F-flotufolastat-avid lesions per miTNM categories. Patient-level detection rates were stratified by: pre-PET scan PSA; absolute/relative difference in PSA (ΔPSA) between negative first and second PET; PSA velocity (PSAvel); PSA doubling time (PSAdt). Receiver operating characteristic (ROC) curve analyses were performed.

**Results:**

Of 101 patients (median age = 70 years; median PSA = 0.79 ng/mL), 58 (57%) had a positive second ^18^F-flotufolastat scan. Detection rates increased with PSA level, ΔPSA, PSAvel, and PSAdt. Differences in PSA level, ΔPSA, PSAvel, and PSAdt between groups with/without suspicious ^18^F-flotufolastat uptake were statistically significant (*p* < 0.05). Cut-offs for statistically optimal trade-off between negative and positive second PET (ROC analyses) were: PSA level > 0.82 ng/mL; absolute ΔPSA > 0.50 ng/mL; relative ΔPSA > + 100%; PSAvel > 0.30 ng/mL/y; PSAdt ≤ 17 months.

**Conclusion:**

In over half of patients presenting with BCR after radical prostatectomy and an initial negative ^18^F-flotufolastat PET, second ^18^F-flotufolastat PET identified recurrent lesions. PSA level at time of second PET, absolute/relative ΔPSA, PSAvel, and PSAdt influenced scan positivity.

## Introduction

Prostate cancer (PCa) is the second most common cancer among men worldwide, with around 1.5 million new cases in 2022 [1]. Up to 30% of men experience biochemical recurrence (BCR) of PCa after initial curative-intent treatment, which poses considerable clinical challenges [2, 3]. An early detection of recurrent lesions is essential for identifying patients suitable for local treatment options, for example, localised salvage therapy.

In the last decade, conventional imaging has increasingly been replaced by radiolabelled prostate‐specific membrane antigen (PSMA)-targeted positron emission tomography (PET) in this setting [4]. PSMA PET is considered the most sensitive and precise diagnostic tool for detection of BCR in patients with PCa [5, 6].

The detection rates have been shown to increase with increasing prostate-specific antigen (PSA) for all approved PSMA-targeting PET radiopharmaceuticals [7–9] and emerging clinical data have demonstrated a promising diagnostic performance of ^18^F-flotufolastat even at very low (< 0.2 ng/mL) PSA levels [10]. However, patients with BCR of PCa may still present with a negative PSMA PET scan [11, 12].

In a preliminary study by Thin et al., follow-up PSMA PET scans using ^68^Ga-PSMA-11 were highly efficient in detecting PCa lesions in patients with BCR despite negative baseline PSMA PET scan without in-depth analysis of detection efficacy for different subgroups of PSA level and PSA kinetics [13]. However, no studies evaluating follow-up ^18^F-flotufolastat PET imaging after a negative PET in patients with recurrent PCa have been conducted so far.

The aim of this retrospective analysis was to investigate the potential of ^18^F-flotufolastat PET for detection and localisation of recurrent disease after previous negative staging with ^18^F-flotufolastat PET, with special focus on the influence of PSA level and PSA kinetics, to identify PSA parameters with significant influence on tumour detection efficacy in patients with prior negative PSMA PET.

## Methods

### Patients

All included patients received radical prostatectomy with curative intent as primary therapy and underwent a ^18^F-flotufolastat PET scan for suspected BCR of PCa (PSA ≥ 0.2 ng/mL) which did not show a correlate for the rising PSA level and was therefore considered PSMA-negative. These patients then received a second PET scan at our institution between 2019 and 2024. The data from these patients were retrospectively reviewed. Patients who received further prostate-specific therapy, such as androgen deprivation therapy or radiation therapy, were excluded from the study, regardless of whether this further therapy took place before the first PET scan for suspected BCR or between the first PET and further PET scans. All reported investigations were conducted in accordance with the Declaration of Helsinki and with national regulations. This retrospective analysis was approved by the local Ethics Committee (permit 99/19) and the requirement to obtain informed consent was waived. The administration of ^18^F-flotufolastat complied with The German Medicinal Products Act, AMG §13 2b, and the responsible regulatory body (Government of Oberbayern).

### ^18^F-Flotufolastat

^18^F-Flotufolastat was synthesised as previously described [14]. A median activity of 264 MBq of ^18^F-flotufolastat (range 122–440 MBq) was administered as an intravenous bolus at a median time of 65 min (range 56–125 min) before scanning. All patients received contrast-enhancing agents.

The patients in the study underwent either PET/CT using a Biograph mCT flow or a Biograph Vision scanner (Siemens Healthineers, Erlangen, Germany), or PET/MRI using an integrated whole-body Siemens Biograph mMR PET/MRI system (Siemens Healthineers Erlangen, Germany), as described previously [15, 16].

All PET scans were acquired in 3-dimensional mode with an acquisition time of 3–4 min per bed position, or 1.1–1.5 mm/second using flow technique. Emission data were corrected for randoms, dead time, scatter, and attenuation, and were reconstructed iteratively by an ordered-subsets expectation maximisation algorithm (True X, 4 iterations, 8 subsets), followed by a post-reconstruction smoothing Gaussian filter (3 mm full width at half maximum).

### Image analysis

All follow-up PET scans were reviewed by an experienced, board‐certified nuclear medicine physician (Isabel Rauscher) and an expert board-certified radiologist (Sonja Kirchhoff), both blinded to the patients’ medical histories. In patients with a negative second PET, up to three further ^18^F-flotufolastat PET scans were analysed, if available. Any focal tracer uptake higher than the surrounding background without association to physiological uptake was considered suspicious of PCa recurrence. All suspicious lesions were classified per miTNM V2 classification [17] as follows: (a) miTr: local recurrence (former prostatic bed), (b) miN1/2 or miM1a: pelvic or extrapelvic lymph node metastases, (c) miM1b: bone metastases, and (d) miM1c: visceral metastases (for example, lung, liver). Typical pitfalls in the evaluation of PSMA PET scans such as low-to-moderate PSMA expression due to osteoblastic changes (i.e. fractures or degenerative changes) were taken into account [18].

### Statistical analysis

All statistical analyses were conducted with MedCalc software (version 13.2.0, 2014; MedCalc, Ostend, Belgium). The detection rate was determined for the second PET scan and stratified by pre-scan PSA (ng/mL), absolute and relative difference in PSA level (∆PSA) between the negative first scan and the second scan, PSA kinetics (PSA velocity [PSAvel], ng/mL/y; PSA doubling time [PSAdt], months), and tumour localisation. PSAvel, and PSAdt were calculated using a calculation tool of the Memorial Sloan Kettering Cancer Center [19]. Mann–Whitney U tests were used to evaluate differences of PSA level, ΔPSA (absolute/relative), PSAvel, and PSAdt between groups with and without pathological uptake. All tests were performed two-sided with a significance level of α = 5%. Separate receiver operating characteristic (ROC) curve analyses including calculation of the area under the curve (AUC) were performed for all parameters. In addition to these univariate analyses, a multivariate regression model was created using the logarithms of PSA level, PSAdt and relative ΔPSA. The Youden indices (calculated as the sensitivity + specificity – 1) were determined to identify the best cut-off values of PSA level, ΔPSA (absolute/relative), PSAvel, and PSAdt for optimising the combination of sensitivity and specificity.

## Results

### Patients

**Fig. 1:**
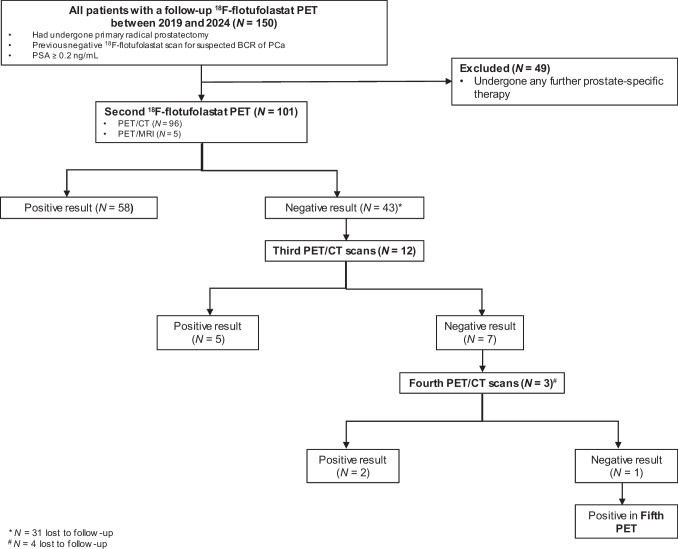
Flowchart illustrating the numbers of patients receiving ≥ 1 follow-up ^18^F-flotufolastat PET scan following an initial negative scan

Data from 101 patients were included in this retrospective analysis. The patients had a median age of 70 (range 49–83) years and a median pre‐scan PSA level of 0.79 (range 0.17–9.44) ng/mL prior to the second PET scan. Of the 43 patients with negative second PET after initial negative baseline PET, nine, two, and one received a total number of three, four, and five PSMA PET scans, respectively (for details see Fig. 1).

The median time interval between the initial and the second scan was 10.7 (range 3.4–58.6) months for all patients (Table 1). The majority of second PET examinations were PET/CT scans (*n* = 96/101; 95%) while only 5/101 (5.0%) patients received PET/MRI examinations. All further follow-up PET examinations were PET/CT scans.

**Table 1:** Baseline demographics and patient characteristics

		***N* = 101**
**Age at second PET scan after surgery in years, median (range)**		70 (49–83)
**ISUP Grade Group, n (%)**	I II III IV V Unknown	10 (9.9) 32 (32) 35 (35) 9 (8.9) 9 (8.9) 6 (5.9)
**pT, n (%)**	pT2	50 (50)
	pT3	47 (47)
	Unknown	4 (4.0)
**pN, n (%)**	pN0 pN1 Unknown	77 (76) 14 (14) 10 (9.9)
**Positive margin, n (%)**	R0	60 (59)
	R1	22 (22)
	Rx	6 (5.9)
	Unknown	13 (13)
**PSA nadir in ng/mL, n (%)**	< 0.1 0.1–0.2 > 0.2 Unknown	64 (63) 6 (5.9) 12 (21) 19 (19)
**Number of PET scans per patient, n (%)**	2 3 4 5	89 (88) 9 (8.9) 2 (2.0) 1 (1.0)
**Injected activity in MBq, median (range)**		264 (122–440)
**Uptake time in min, median (range)**		65 (56–125)
Time from first (negative) to second PET scan in months, median (range)*		10.7 (3.4–58.6)
**Last PSA value prior first PET scan in ng/mL, median (range)**		0.37 (0.10–4.49)
**Last PSA value prior second PET scan in ng/mL, median (range)**		0.79 (0.17–9.44)

ISUP, International Society of Urological Pathology; PET, positron emission tomography; pN, pathological node status; PSA, prostate-specific antigen; pT, pathological tumour size

### Detection rates and tumour localisation

The second PET scans revealed ≥ 1 localised area suspicious for recurrent disease in 58/101 patients (57%). Detection rates of ^18^F-flotufolastat PET increased with pre-scan PSA from 46% (*n* = 13/28; 95% CI = 0.275–0.661) at PSA ≤ 0.5 ng/mL to 47% (*n* = 18/38; 95% CI = 0.310–0.642) at PSA > 0.5–1 ng/mL, 72% (*n* = 18/25; 95% CI = 0.506–0.879) at PSA > 1–2 ng/mL, and 90% (*n* = 9/10; 95% CI = 0.555–0.998) at PSA > 2 ng/mL. An increase of detection rates was also observed with increasing ∆PSA as follows: 43% (*n* = 28/65; 95% CI = 0.309–0.560) for ∆PSA ≤ 0.5 ng/mL, 74% (*n* = 17/23; 95% CI = 0.516–0.898) for ∆PSA > 0.5–1 ng/mL, and 100% (*n* = 13/13; 95% CI = 0.753–1.000) for ∆PSA > 1 ng/mL. A similar trend was observed for PSAvel with persistently high detection rates for PSAvel > 1 ng/mL/y. Detection rates increased with shorter PSAdt, with the exception of a small decrease from 78% (*n* = 7/9; 95% CI = 0.400–0.972) for PSAdt > 4–6 months to 67% (*n* = 6/9; 95% CI = 0.299–0.925) at PSAdt ≤ 4 months.

**Fig. 2:**
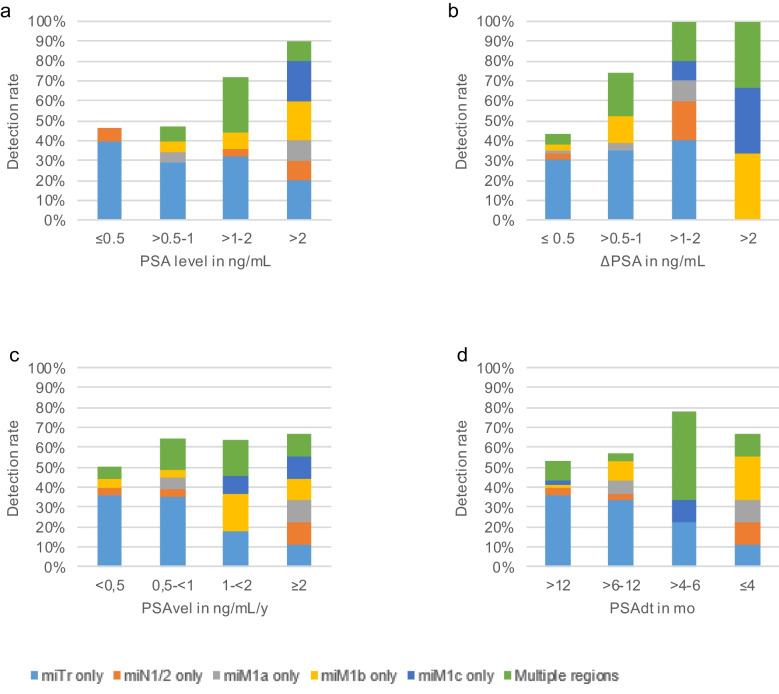
Overall detection rate of the second ^18^F‐flotufolastat PET scan in relation to pre-scan PSA (**a**), change in PSA level (ΔPSA) (**b**), PSA velocity (PSAvel) (**c**), and PSA doubling time (PSAdt) (**d**) miM1b, bone metastases; miM1c: visceral metastases, miN1/2/miM1a, pelvic or extrapelvic lymph node metastases; miTr, local recurrence; PET = positron emission tomography; PSA = prostate-specific antigen

A shift in tumour localisation from predominantly solitary local recurrences to an increase of metastatic disease and multiple lesions was observed with rising PSA level, ∆PSA, and PSAvel, and with decreasing PSAdt. For full details, please refer to Fig. 2** a–d**.

**Fig. 3:**
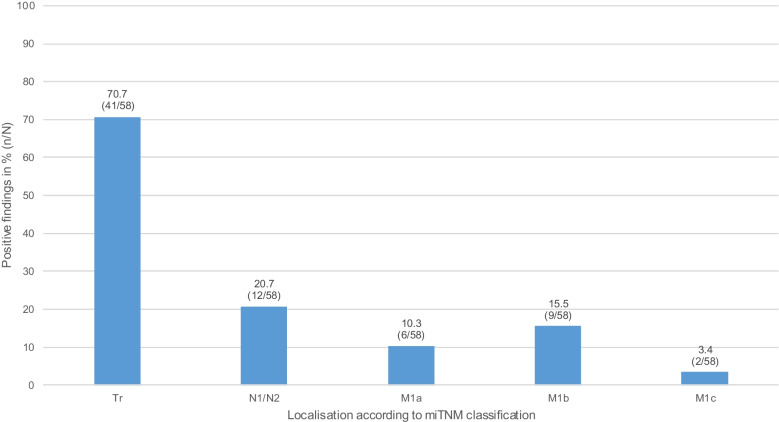
Tumour localisation (region-level detection) with ^18^F‐flotufolastat PET by miTNM classification [17] M1b, bone metastases; M1c: visceral metastases, N1/2/M1a, pelvic or extrapelvic lymph node metastases; PET, positron emission tomography; Tr, local recurrence

A summary of lesion localisation in patients with positive second PET scans is provided in Fig. 3. Per miTNM tumour localisation classification, most suspicious lesions were local recurrence (*n* = 41/58; 71%), followed by pelvic lymph node metastases (*n* = 12/58; 21%). Multiple lesion localisations within one patient were possible.

**Fig. 4:**
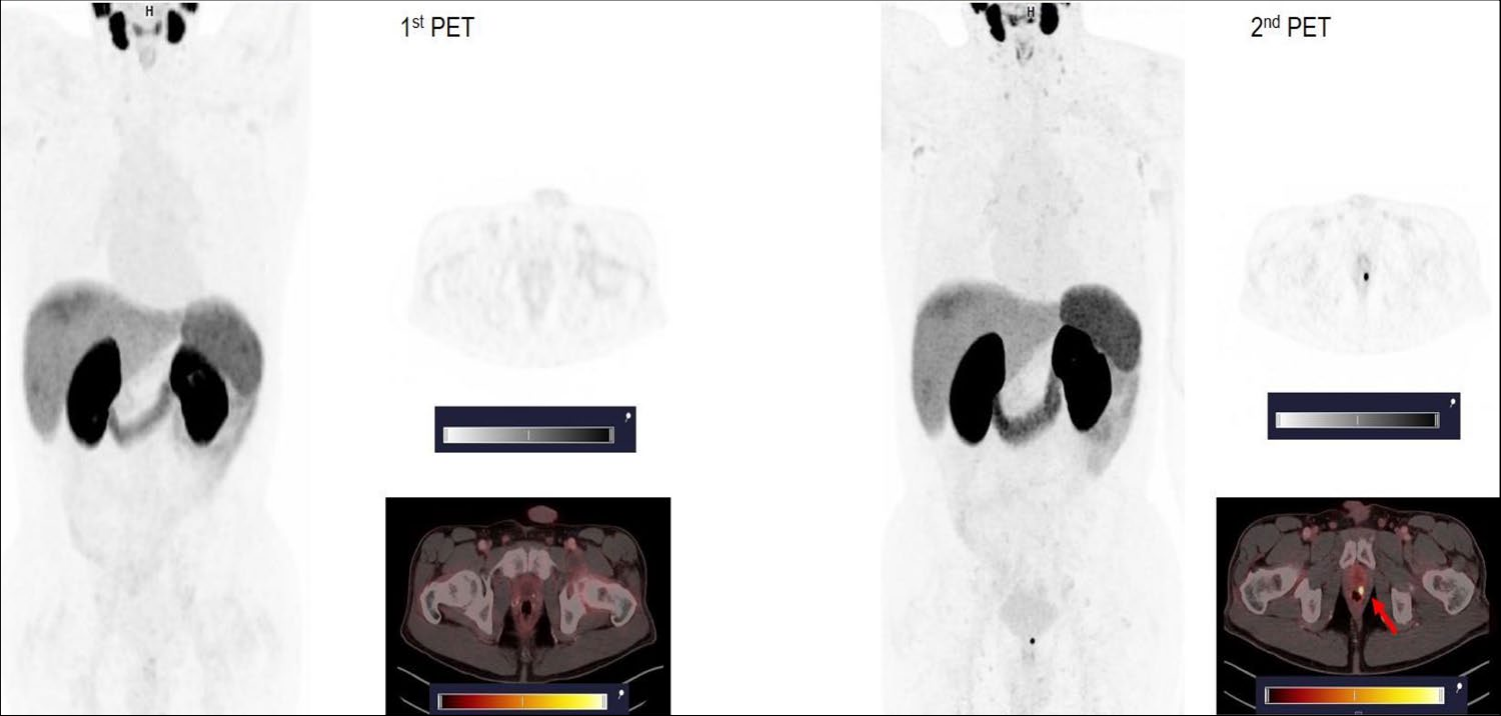
63-year-old patient (pT2c; pN0; Gleason Score = 7a; iPSA = 5.7 ng/mL) presenting with BCR after radical prostatectomy. PSA was undetectable after surgery (< 0.07 ng/mL), then increased to 0.53 ng/mL 2.5 years after surgery. The first ^18^F-flotufolastat PET scan showed negative results. One year after the first PSMA PET scan, his PSA level increased again to 1.1 ng/mL (ΔPSA = 0.57 ng/mL/+ 108%; PSAvel = 0.6 ng/mL/y; PSAdt = 11.0 mo). The second ^18^F-flotufolastat PET was positive, detecting a local, intensively PSMA-expressing recurrent lesion

**Fig. 5:**
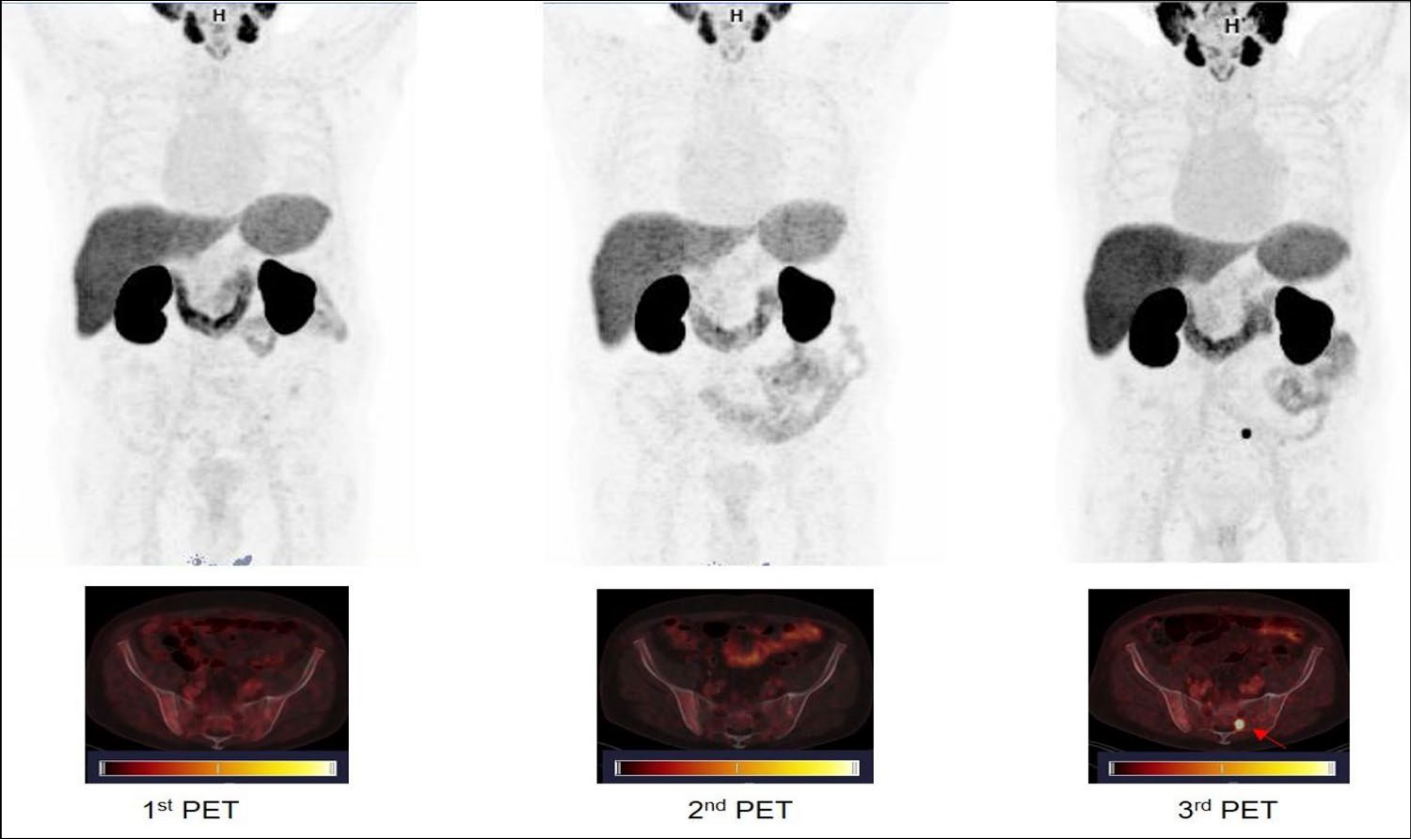
77-year-old patient (pT2b; pN0; pM0; R0; iPSA = 14.96 ng/mL) presenting with BCR after radical prostatectomy. The patient had undetectable PSA after prostatectomy (< 0.07 ng/mL) and began to have a slow, continuous rise in PSA levels starting 7 years after surgery. PSA level increased to 1.08 ng/mL 16 years after surgery, however, a first ^18^F-flotufolastat PET scan was negative. In the 15 months following the first PET scan, PSA level increased again to 1.40 ng/mL (ΔPSA = 0.32 ng/mL), and a second ^18^F-flotufolastat PET was performed which was negative again. Seventeen months after the second PET scan, the patient’s PSA level was 8.74 ng/mL, which corresponded to a ΔPSA of 7.34 ng/mL and a change of + 524% between the second and third PSMA PET scans. PSAdt and PSAvel, respectively, were 6.6 months and 5.1 ng/mL/y based on PSA kinetics between the second and third PSMA PET scans, and were 10.5 months and 2.9 ng/mL/y based on PSA changes across all 3 PSMA PET scans. The third PSMA PET scan showed intensively PSMA-expressing osseous metastases in left massa lateralis, which aligned with the strong PSA increase observed over 17 months

An example of a patient with local recurrence is shown in Fig. 4. Figure 5 illustrates a patient example with a singular bone metastasis.

### PSA level and PSA kinetics in patients with positive versus negative second PET

There were significant differences in PSA level, ΔPSA (absolute and relative), PSAvel, and PSAdt between groups with and without pathological uptake of ^18^F-flotufolastat (*p* < 0.05). For example, patients with positive second PET presented with a median PSA level of 0.97 ng/mL and ∆PSA of 0.52 ng/mL, compared with 0.72 ng/mL and 0.26 ng/mL, respectively, for patients with a negative second PET (for full details, please refer to Table 2). Notably, time to second PET was not relevantly different between patients with a positive versus a negative finding (10.6 months, range 3.4–58.6 versus 10.8 months, range 3.6–44.8).

**Table 2:** PSA level and PSA kinetics in patients with BCR of PCa with positive versus negative findings in the second ^18^F-flotufolastat PET

Parameter	Positive findings (95% CI)	No findings (95%CI)	Two-tailed *P*-value	AUC (95% CI)
Pre-scan PSA, ng/mL	0.97 (0.75–1.15)	0.72 (0.50–0.80)	0.0046	0.665 (0.565–0.765)
Absolute ΔPSA, ng/mL	0.52 (0.32–0.68)	0.26 (0.20–0.34)	0.0013	0.688 (0.588–0.777)
Relative ΔPSA in %	+ 107.9 (67.8–140.0)	+ 78.3 (52.0–88.0)	0.0064	0.659 (0.558–0.751)
PSAvel, ng/mL/y	0.50 (0.40–0.60)	0.30 (0.10–0.44)	0.0030	0.672 (0.572–0.762)
PSAdt, months	11.80 (8.61–13.98)	18.80 (10.62–26.94)	0.0214	0.634 (0.533–0.728)

AUC, area under the curve; BCR, biochemical recurrence; PSA, prostate-specific antigen; PCa, prostate cancer; PSAdt, PSA doubling time; PSAvel, PSA velocity

### ROC curve analysis

**Fig. 6:**
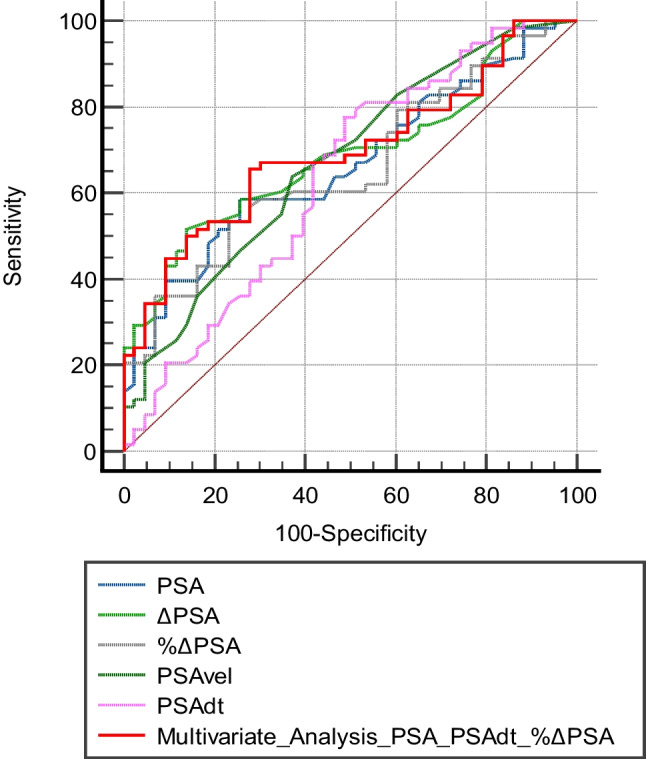
ROC analyses for PSA level, ∆PSA absolute, ∆PSA relative (%∆PSA), PSA velocity (PSAvel), PSA doubling time (PSAdt) and multivariate ROC analysis with PSA level, PSAdt and %∆PSA. PSA, prostate-specific antigen; ROC, receiver operating characteristic

The best trade-off for classification of positive and negative scans was determined by estimating the following cut-off values: PSA level > 0.82 ng/mL (AUC = 0.67), absolute PSA change (ΔPSA absolute) > 0.50 ng/mL (AUC = 0.69), relative PSA change (ΔPSA relative) > + 100% (AUC = 0.66), PSAvel > 0.30 ng/mL/y (AUC = 0.67), and PSAdt ≤ 17 months (AUC = 0.63). The ROC curves for all PSA parameters are presented in Fig. 6.

A multivariate model with the parameters PSA level, PSAdt and ΔPSA relative was statistically significant (*p* = 0.0033) and delivered the ROC curve indicated by the red line in Fig. 6, with an AUC of 0.70.

### Further follow-up PET scans

Most patients (*n* = 89/101; 88%) received only two PSMA PET scans, – i.e., an initial negative scan followed by one follow-up scan. However, for 43% (*n* = 43/101) of those patients, the second PET scan was negative, so that 12 out of these 43 patients underwent at least one further follow-up scan. In 67% (*n* = 8/12) of the patients, a PET-positive lesion was observed in the third to fifth PET scan (for details see Fig. 1). According to the miTNM classification V2, miTr, miN1/2, miM1a, and miM1b was observed in four, three, one, and five patients, respectively.

## Discussion

The results of our retrospective analysis showed that a second PET with ^18^F-flotufolastat detected lesions indicative for recurrent disease in more than half of the patients (*n* = 58/101, 57%) after a first negative ^18^F-flotufolastat PET scan. Important factors that influenced ^18^F-flotufolastat PET positivity were PSA level at the time of the second PET scan, the absolute and relative difference in PSA level between the first and second PET scans, PSAvel, and PSAdt. Notably, the time between first and second PET scan was not a relevant factor for PET positivity.

To date, two studies have investigated the detection rates of follow-up PSMA PET scans after an initial negative PET: Thin et al. determined a slightly lower, but similar overall detection rate of 53% (*n* = 44/83) in the second PET using ^68^Ga-PSMA-11, despite a higher pre-scan PSA level in their study (PSA level 1.6 ng/mL versus 0.79 ng/mL in our analysis) [13]. In a retrospective study by Mjaess et al. using the radiopharmaceutical ^18^F-DCFPyL, a substantially lower detection rate of 41% (*n* = 12/29) was observed [20]. The lower detection rates obtained by Mjaess et al. could be due to the shorter time interval between the PETs: While the median time interval between the first and second PET was 11 months both in our study and in the study by Thin et al., a second PET was already performed after a median of 6 months after the first PET in the study by Mjaess et al.. However, in our analysis, the time interval between PET scans was not a relevant predictor of PET positivity. Another possible reason is that 15/29 patients examined by Miaess et al. received adjuvant radiation therapy after primary therapy. Apart from that, the tracer may also have had an influence on the different detection rate: In a systematic review by Rais-Bahrami et al. comparing the three FDA approved tracers ^18^F-flotufolastat, ^68^Ga-PSMA-11 and ^18^F-DCFPyL in patients with recurrent PCa, higher sample-weighted mean detection rates were found for ^18^F-flotufolastat (79%) than for ^68^Ga-PSMA-11 (71%) and ^18^F-DCFPyL (66%) [21]. Especially at PSA levels < 1 ng/mL, more lesions could be detected using ^18^F-flotufolastat (66%) in relation to ^68^Ga-PSMA-11 (53%) or ^18^F-DCFPyL (42%). In an exploratory analysis of the SPOTLIGHT study by Lowentritt et al., ^18^F-flotufolastat PET also proved to be particularly sensitive in patients with biochemical recurrence and low PSA levels: In patients with PSA < 0.5 ng/mL, ^18^F-flotutolastat PET detected PSMA-positive lesions in almost two-thirds of patients (64%), significantly more than in comparable studies with ^68^Ga-PSMA-11 (38%) and ^18^F-DCFPyL (36%) [7, 9, 10]. The high detection rates for ^18^F-flotufolastat compared to other PSMA tracers are associated with its favourable biodistribution profile, sustained plasma bioavailability, limited urinary bladder activity, high-affinity receptor binding and internalization [22–25].

The mean effective dose for ^18^F-flotufolastat PET imaging (0.0141 mSv/MBq) [22] is slightly lower than the mean effective dose specified for PET imaging with other tracers such as ^68^Ga-PSMA-11 (0.0158 mSv/MBq) [26], ^18^F-DCFPyL (0.0165 mSv/MBq) [27] and ^18^F-PSMA-1007 (0.0220 mSv/MBq) [28]. For an applied activity of 300 MBq ^18^F-flotufolastat, the mean effective dose is 4.2 mSv. This radiation exposure is moderate compared to other imaging modalities and may justify performing repetitive ^18^F-flotufolastat PET examinations when indicated [22, 29]. In our study, an even lower median activity of 264 MBq (range: 122–440 MBq) was administered, which corresponds to an effective dose of 3.7 mSv (range 1.7 mSv–6.2 mSv).

Detection rates increased with increasing PSA level as known from the common literature for ^18^F-flotufolastat and several other PET radiopharmaceuticals [8, 15, 30, 31]. Considering PSA kinetics, our detection rates increased with larger (absolute/relative) changes in PSA level, greater PSAvel, and shorter PSAdt between the first negative and the second PET scan. Differences in PSA level, ΔPSA (absolute and relative), PSAvel, and PSAdt between patients with positive versus negative second PET scans were statistically significant. Our ROC analysis revealed > 0.82 ng/mL for PSA level at the time of the second PET, > 0.50 ng/mL for ΔPSA absolute, > + 100% for ΔPSA relative, PSAvel > 0.30 ng/mL/y, and PSAdt ≤ 17 mo as the statistically optimal trade-off between a negative and a positive scan based on the calculation of the Youden indices.

Similar to our findings, Thin et al. observed statistically significant differences in PSA level, ΔPSA (absolute and relative), PSAvel, and PSAdt between patients with positive versus negative second PET scans (with ^68^Ga-PSMA-11). However, they indicated higher cut-off values despite similar AUCs (range: 0.64–0.70 vs. 0.63–0.69 in our analysis): 4.80 ng/mL for PSA level, 0.95 ng/mL/290% for absolute/relative ΔPSA, 0.033 ng/mL/week (≈ 1.7 ng/mL/y) for PSAvel, and 7.91 mo for PSAdt [13]. A possible explanation for these differences could be that the majority of the patients (*n* = 51/83; 61%) analysed by Thin et al. received salvage therapy before the initial negative PET scan and some patients received radiation therapy as primary therapy. In our retrospective analysis, only patients with BCR after radical prostatectomy without any other prostate-specific therapy were included. In addition, our cut-offs (with the exception of the cut-off for ∆PSA absolute) corresponded to higher sensitivities (range: 52–78%, median = 59%) than the cut-offs used by Thin et al. (range: 34–71%, median = 57%).

On the contrary, Mjaess et al. could not identify a statistically significant effect of pre-scan PSA level or PSAdt on the positivity of the second PSMA PET after an initial negative PET scan with ^18^F-DCFPyL in patients with recurrent PCa. However, this might be due to the small number of patients (*n* = 29) included in their study [20]. Further studies to explore the relationship of PSA kinetics with PSMA PET positivity and, furthermore, defining predictors of BCR, would be of interest.

Remarkably, our analysis revealed local recurrence in 41/58 patients (71%) with a positive second PET scan. This is a relevant difference to the proportions described in other studies such as Rauscher et al. who evaluated recurrence localisation in patients with early BCR. Rauscher et al. detected local recurrence in only 69/176 patients (39%) with positive ^68^Ga-PSMA-11 PET [32]. A possible reason for this difference might be the pre-selection of our cohort including only patients with prior negative PET, potentially increasing the number of slowly growing local recurrences rather than rapidly growing metastases.

In our analysis, 12 out of 43 patients (28%) with a negative second PET scan underwent at least one further follow-up PSMA PET scan. Interestingly, 67% (*n* = 8/12) presented with a positive lesion in one of these further follow-up PSMA PET scans. While it is difficult to draw conclusions from a sample of only 12 patients, these results suggest that even after two negative PET scans, further follow-up PSMA PET scans might be beneficial for the detection of recurrent PCa lesions.

About 5–10% of prostate cancers do not overexpress PSMA which might be a possible explanation for the negative results at the follow-up PET scans [12, 33, 34]. Using a non-PSMA-targeting radiopharmaceutical, such as ^18^F-fluciclovine which targets upregulated amino acid transporters in metabolising cancer cells [30, 35] could be a more suitable option for follow-up scans in such individuals. Overall, fluciclovine PET was shown to be less sensitive in the detection of recurrent PCa lesions after radical prostatectomy than PSMA PET in a prospective trial by Calais et al.. However, fluciclovine PET was able to detect lesions that remained undetected in PSMA PET in a subset of patients [36]. A prospective study to explore the potential of ^18^F-fluciclovine after a negative PSMA PET is planned (NCT06859203).

In our study, the time interval between the first negative and the second scan did not significantly differ between patients with positive and negative second PET. Patients with a positive second PET had a similar time interval between the PET scans as patients with a negative second PET (10.6 versus 10.8 months, respectively). It is more likely that crucial factors are absolute PSA levels and PSA kinetics. Pre-scan PSA level > 0.82 ng/mL, ∆PSA > 0.50 ng/mL/+ 100%, PSAvel > 0.30 ng/mL/y, and PSAdt ≤ 17 months represent guidance for timing a second PET scan.

Our study provides potential clinical thresholds for the indication of a second PSMA PET scan. However, there is only moderate discriminatory ability as indicated by the AUCs ranging from 0.63 to 0.69. The AUC of the ROC curve corresponding to the multivariate model using the logarithms of PSA level, PSAdt and relative ∆PSA is only slightly higher (AUC = 0.70) and the added value of the model therefore moderate.

As a potential limitation of our study, it should be acknowledged that the retrospective nature of our analysis limits the broader applicability of our data. It was not known whether positive lesions were confirmed as true positives through histopathology. In most patients, no further follow-up imaging was performed for lesion validation. This potentially leads to an overestimation of the detection rate across PSA levels. However, reassuringly recent clinical data with ^18^F-flotufolastat show a patient-level positive predictive value of 82% (95% CI = 71.2%–90.5%) in patients with histopathological data [8]. Further studies with ^18^F-flotufolastat, ^18^F-rhPSMA-7 and ^68^Ga-PSMA-11 describe even higher positive predictive values ranging from 88 to 95% [37–39]. For most patients with positive findings in the second PET, information about treatment changes was missing. In a study by Rauscher et al., a ^18^F-flotufolastat PET scan in patients with BCR of PCa after radical prostatectomy, led to a potential change in treatment plans in 63% of patients, thus suggesting a high clinical impact of ^18^F-flotufolastat PET imaging on patient management [40]. Furthermore, 18 patients presented with PSA nadir ≥ 0.1 ng/mL, considered as PSA persistence, and for 19 patients PSA nadir was not available, potentially influencing our results. In addition, the wide range of uptake times (56–125 min) could limit the comparability of the PET scans evaluated in our study, although tumour uptake of ^18^F-rhPSMA-7 has been shown to be stable across different ranges of uptake times by Oh et al. [41].

## Conclusion

In summary, our study demonstrated that following a negative ^18^F-flotufolastat PET scan in patients with suspected BCR of PCa after radical prostatectomy, a second ^18^F-flotufolastat PET scan could detect tumour lesions in more than half of the patients. Important factors that influenced positivity were PSA level at the time of the second PET, the absolute and relative difference in PSA level between the first and second PET scans, PSAvel, and PSAdt.

## Data Availability

The datasets generated during and/or analysed during the current study are available from the corresponding author on reasonable request.
